# Sexualité des femmes ménopausées en Afrique sub-saharienne: exemple du Sénégal

**DOI:** 10.11604/pamj.2019.32.1.17755

**Published:** 2019-01-01

**Authors:** Abdoul Aziz Diouf, Djibril Diallo, Papa Youssou Niang, Aminata Niass, Cyr Espérance Koulimaya-Gombet, Moussa Diallo, Jean Charles Moreau, Alassane Diouf

**Affiliations:** 1 Service de Gynécologie du Centre Hospitalier National de Pikine, Sis Camp de Thiaroye, Dakar, Sénégal; 2 Clinique Gynécologique et Obstétricale de l’Hôpital Aristide Le Dantec, Dakar, Sénégal

**Keywords:** Ménopause, Sexualité, Sècheresse vaginale, Sénégal, Menopause, sexuality, vaginal dryness, Senegal

## Abstract

**Introduction:**

En Afrique, la vie sexuelle après la ménopause reste un domaine mal exploré du fait de son caractère tabou. L'objectif de cette étude était d'apprécier la qualité de la sexualité du couple au cours de la ménopause.

**Methodes:**

Il s'agissait d'une une enquête transversale concernant un échantillon représentatif de 320 femmes ménopausées. Les critères d'inclusion étaient la ménopause naturelle. N'étaient pas incluses de l'étude les patientes ayant fait l'objet d'une ménopause précoce ou iatrogène. La fiche d'enquête était divisée en 4 chapitres : les caractéristiques socio-culturelles de la femme, les données cliniques, les données psycho-sexuelles et les données thérapeutiques. Nous avons utilisé la comparaison des proportions et le test du Chi 2 avec un seuil de signification inférieur à 0,05.

**Resultats:**

L'âge moyen des femmes était de 60 ans. L'âge moyen de survenue de la ménopause était de 48 ans et l'ancienneté de la ménopause était de 11,3 ans. Aucune femme de notre étude n'était épargnée par les manifestations du syndrome climatérique. Les bouffées de chaleur étaient présentes dans 85,9%, la sècheresse vaginale dans 62,8% et les troubles urinaires dans 52,5%. Seules les femmes mariées déclaraient avoir des rapports sexuels avec leurs conjoints (62,1%). Ces rapports sexuels étaient occasionnels dans 68,9% des cas alors que 18,1% des femmes n'avaient plus d'activité sexuelle. La diminution de l'activité sexuelle était due aux troubles de l'érection du conjoint (62% des couples) et au manque de désir sexuel (83,5% des femmes). Une absence d'excitation sexuelle et d'orgasme étaient également retrouvée respectivement dans 92% et 100%. Cependant, 93,5 % des femmes mariées jugeaient supportable leur vécu.

**Conclusion:**

Le statut marital, la dyspareunie, la sècheresse vaginale et les troubles érectiles du conjoint ont un impact réel sur la sexualité de la femme ménopausée au Sénégal.

## Abstract (en)

**Introduction:**

In Africa, sex life after menopause remains an under-explored topic due to the fact that it is a taboo. This study aims to evaluate the quality of couple's sex life during menopause.

**Methods:**

We conducted a cross-sectional survey of a representative sample of 320 postmenopausal women. The inclusion criteria were natural menopause. Patients who had experienced early or iatrogenic menopause were excluded. Investigation form was divided into 4 sections: social and cultural characteristics, clinical data, psycho-sexual data and therapeutic data. Comparison of proportions and chi-squared test with a significance threshold of less than 0.05 were used.

**Results:**

The average age of women was 60 years; the average age of onset of menopause was 48 years and the age of the menopause was 11.3 years. All woman suffered from climacteric syndrome. Hot flushes occured in 85.9% of women, vaginal dryness in 62.8% and urinary disorders in 52.5%. Only married women reported having sex with their partner (62.1%). Women had sex occasionally in 68.9% of cases, while 18.1% of women reported no sexual activity. Decreased sexual activity was due to partner's erectile dysfunction (62% of couples) and the lack of sexual desire (83.5% of women). Lack of sexual appetite and orgasm were also reported in 92% and 100% of cases. However, 93.5% of married women thought their life was bearable.

**Conclusion:**

Marital status, dyspareunia, vaginal dryness and partner's erectile dysfunction have a significant impact on sexuality of menopausal women in Senegal.

## Introduction

En Afrique, la vie sexuelle après la ménopause reste un domaine mal exploré du fait de son caractère tabou. D'autre part, l'impact des troubles climatériques sur la qualité de vie de la femme [1,2] est souvent conditionné par nos mœurs qui sont caractérisées essentiellement par la discrétion et la résignation dans le domaine de la sexualité [3]. Cependant, accepter cette situation serait une injustice à l'égard de ces femmes ménopausées qui ne demandent qu'à être prises en charge de manière globale, en s'appuyant effectivement sur nos réalités socio-culturelles; d'autant plus qu'avec l'influence de la mondialisation culturelle, les craintes et les plaintes des femmes par rapport à la ménopause sont de plus en plus exprimées. L'étude que nous avons voulu mener constitue un point de repère sur la sexualité de la femme ménopausée en milieu africain. L'objectif était d'apprécier la qualité de la sexualité du couple au cours de la ménopause.

## Méthodes

Il s'agissait d'une une enquête transversale réalisée au Centre Médical de l'Institut de Prévoyance Retraite du Sénégal (IPRES) au cours de l'année 2014 et concernant un échantillon représentatif de femmes ménopausées. Les calculs statistiques avaient abouti au chiffre de 288 femmes, auquel nous avons ajouté 10% pour faire face à une éventuelle déperdition en cours d'étude. La taille définitive de l'échantillon était ainsi de 320 femmes ménopausées. Les critères d'inclusion étaient la ménopause naturelle confirmée avec arrêt des règles depuis au moins 1 an. N'étaient pas incluses de l'étude les patientes ayant fait l'objet d'une ménopause précoce, d'une hystérectomie, d'une chirurgie de l'ovaire, d'une chimiothérapie ou d'une radiothérapie en période d'activité génitale. La fiche d'enquête était divisée en 4 chapitres : les caractéristiques socio-culturelles de la femme, les données cliniques, les données psycho-sexuelles et les données thérapeutiques. Nous avons utilisé la comparaison des proportions et le test du Chi 2 avec un seuil de signification inférieur à 0,05.

## Résultats

La moyenne d'âge au moment du recrutement des femmes était de 60±6,8 (extrêmes de 43 et 83 ans). L'ethnie Wolof prédominait largement dans notre série avec 171 cas (53,4%), suivi respectivement des Pulars (19,7%) et des sérères (11,6%). Les femmes actives ne représentaient que 30% de la série, la majorité étant des femmes au foyer 59,4%. La parité moyenne était de 6,4±2,8. Les femmes qui vivaient en couple dans notre série représentaient 62,1% tandis que les 121 femmes restantes étaient célibataires (2,2%), veuves (31%) ou divorcées (4,7%). Parmi les mariées, 54% vivaient dans un foyer polygame. La consommation d'alcool et de tabac était retrouvée dans les mêmes proportions (1,6%). Parmi les foyers polygames, 21,5 % des époux avaient une activité rémunérée et près des quatre cinquièmes bénéficiaient de leurs droits à la retraite. Malgré l'âge avancé des patientes, le sport était pratiqué dans 43 % des cas avec notamment la marche (42%). L'âge moyen de survenue de la ménopause était de 48 ans. Le niveau d'information sur la ménopause était élevé et concernait 82,2% des femmes. Cependant, cette information était souvent incomplète et donnait une perception erronée de la ménopause (53%). L'ancienneté de la ménopause au moment du recrutement était de 11,3 ans. Aucune femme de notre étude n'était épargnée par les manifestations du syndrome climatérique (Tableau 1). Les bouffées de chaleur étaient présentes dans 85,9% des cas. Les autres symptômes étaient dominés par les douleurs ostéo-articulaires (84,1%), les sueurs nocturnes (79,3%), les céphalées (78,4%) et l'insomnie (71%). La sècheresse vaginale et les troubles urinaires à type de dysurie et pollakiurie étaient signalés dans respectivement 62,8% et 52,5% des cas. Seules les femmes mariées déclaraient avoir des rapports sexuels avec leurs conjoints (62,1% des cas), alors que les célibataires, les veuves et les divorcées affirmaient ne plus avoir de rapports sexuels. La fréquence des rapports sexuels pour les 199 femmes qui vivaient en couple était entre 0 et 3 par semaine. Les rapports sexuels étaient occasionnels et ne dépassaient pas une fois par semaine dans 68,9% des cas. Ils étaient supérieurs à 2 fois par semaine dans 13% des cas; et 18,1% des femmes n'avaient plus de rapport sexuel avec leur conjoint (Tableau 2). La diminution du nombre de rapports sexuels était liée d'une part aux conjoints qui avaient des troubles de l'érection (62% des couples), et d'autre part au trouble du désir sexuel exprimé par 83,5% des femmes. Une absence d'excitation sexuelle et d'orgasme étaient également retrouvée respectivement dans 92% et 100%. En dépit de cette situation, 80,6% des patientes affirmaient que les sentiments qui animaient leur union depuis le début n'avaient pas changé. Ainsi, 93,5 % des femmes mariées jugeaient supportable leur vécu et avaient une attitude de résignation face à ces troubles sexuels au sein de leur couple (Tableau 3). Concernant le volet thérapeutique pour palier les troubles de la ménopause, seules 3 femmes (9%) avaient eu un traitement hormonal de la ménopause, alors que 152 d'entre elles (47,5%) avaient eu recours à un tradipraticien.

**Tableau 1:** Fréquence des principaux symptômes rapportés par les femmes ménopausées à Dakar

Symptômes	Fréquence absolue n= 320	Fréquence relative (%)
Bouffées de chaleur	275	85,9
Insomnie	228	71,3
Sueurs nocturnes	253	79,3
Asthénie	222	69,4
Céphalées	251	78,4
Arthralgies	269	84,1
Nervosité	172	53,8
Troubles urinaires	168	52,5
Sècheresse vaginale	201	62,8

**Tableau 2:** Nombre de rapports sexuels pour les femmes vivant en couple

Rapports sexuels	Effectif (n = 199)	Pourcentage (%)
Aucun	36	18,1
Une fois par 3 mois	39	19,6
Une fois par mois	28	14,1
Une fois par 15 jours	37	18,6
Une fois par semaine	33	16,6
Deux fois par semaine	19	9,5
Plus de deux fois par semaine	7	3,5

**Tableau 3:** Qualité de la vie sexuelle des femmes ménopausées à Dakar

	Effectif (n = 320)	Pourcentage (%)
**Désir sexuel**		
Parfois	60	18,7
Absente	260	81,3
**Excitation sexuelle**		
Parfois	16	8
Absentes	183	92
**Pas de rapport sexuel**		
Orgasme	121	-
Parfois	0	0
Absent	199	100
**Pas de rapport sexuel**		
Vécu psycho-sexuel	121	-
Satisfaisant	7	3,5
Supportable	186	93,5
Insupportable	6	3
Pas de rapport sexuel	121	-

## Discussion

Notre étude nous permet d'avoir un profil général de la sexualité de la femme sénégalaise ménopausée qui se traduit par une moyenne d'âge de 60 ans, une présence du syndrome climatérique avec des troubles trophiques vulvo-vaginaux, une cessation des rapports sexuels chez les femmes divorcées, veuves ou célibataires du fait du poids socio-culturel et religieux, et une réduction considérable de la fréquence des rapports sexuels chez celles qui vivent en couple. Cette réduction de la fréquence des rapports sexuels est souvent due à la dyspareunie qui affecte 62% des couples, ou alors au conjoint qui présente un dysfonctionnement érectile (62% des couples). Ce dernier point reste prévisible dans la mesure où dans cette génération, la différence d'âge de 15 à 20 dans le couple, en faveur du conjoint, est souvent la règle ; les femmes de 60 et plus ont fréquemment des conjoints âgés de 75 à 80 ans en moyenne présentant naturellement un dysfonctionnement érectile. Malgré ces troubles de la sexualité qu'elles jugent souvent supportables, elles ne consultent pas pour une amélioration de leur qualité de vie et préfèrent garder une attitude de résignation ou alors s'en remettre à la médecine traditionnelle. Les résultats d'une enquête sénégalaise en 2006 [1] sur le vécu de la ménopause montraient pratiquement ce même profil.

Il ne serait pas raisonnable de nier les mutations de la sexualité des femmes africaines au cours de ces dernières décennies [4], mais ces mutations ne sont pas encore parvenues à libérer les couples de ces comportements pudiques et de ces sujets tabous qui ont toujours caractérisés leur intimité. Ces mutations sont d'autant plus absentes que le couple est âgé et vivant en zone semi-urbaine ou rurale. Le manque de communication du couple sur leur sexualité et l'attitude de résignation des femmes ménopausées face aux conséquences sexuelles du déficit hormonal (sècheresse vaginale, dyspareunie) constituent les principaux freins d'une sexualité épanouie et assumée. Rappelons que toutes les femmes qui vivaient en couple (199 femmes) se plaignaient de sècheresse vaginale et que 69,3% d'entre elles (138 femmes) rapportaient une dyspareunie. Cette même attitude est retrouvée dans les pays maghrébins notamment le Maroc et la Tunisie; en effet, une étude réalisée par Kadri et al [5,6] sur un échantillon de 100 femmes ménopausées (Casablanca et Tunis) retrouvait un âge moyen de 50 ans, une baisse de l'activité sexuelle chez 86,5 % des marocaines et 63,3 % des tunisiennes. Les causes recueillies auprès de l'échantillon marocain étaient essentiellement une dyspareunie (8,9 %), une sécheresse vaginale (17,80 %) alors que pour l'échantillon tunisien la dyspareunie a été rapportée par 5,5 % des femmes et la sécheresse vaginale par 16,70 % des femmes.

En réalité, ce comportement de désengagement des femmes ménopausées face à leur sexualité s'explique par le fait que cette génération a grandi dans une période où il n'y avait aucune valorisation du sexe qui est d'ailleurs programmé pour disparaître avec l'âge. Cette conception reste cependant désuète pour cette nouvelle génération de femmes qui lorsqu'elles atteindront l'âge de la ménopause, auront une attitude différente de celle de leurs ainées sur la sexualité. C'est d'ailleurs ce constat qui s'affirme dans les pays occidentaux où les femmes de 60 ans et plus affirmaient dans beaucoup de travaux [7-9], être toujours intéressées par l'activité sexuelle. Car après tout, le vieillissement c'est dans la tête ! Colson [10], sur son article intitulé « la sexualité après 60 ans, déclin ou nouvel âge de vie ?» rapportait dans ses conclusions « Nous n'avons pas le choix face au vieillissement physiologique, mais il nous reste celui de notre vie face aux limitations imposées par l'âge. Notre choix de vieillesse sera notre dernier choix de vie. Elle pourra être, selon les cas, décompensée, surcompensée ou optimisée ».

## Conclusion

Le statut marital, la dyspareunie et la sècheresse vaginale ont un impact réel sur la sexualité de la femme ménopausée au Sénégal. A cela s'ajoute les troubles érectiles du conjoint secondaire à la vieillesse. L'attitude de résignation face à ses troubles n'est en réalité que le résultat d'une profonde structuration de la société où la sexualité n'a vraiment pas une place prépondérante.

### Etat des connaissances actuelles sur le sujet

- La sexualité des femmes ménopausées est un tabou en Afrique sub-saharienne, particulièrement au Sénégal;
- Les femmes consultent rarement pour les troubles vasomoteurs à la ménopause, encore moins pour des problèmes de sexualité.

### Contribution de notre étude à la connaissance

- Les troubles de la sexualité chez les femmes africaines ménopausées sont une réalité;
- Les femmes gardent souvent une attitude de résignation face aux troubles sexuels de la ménopause;
- Le statut marital, la dyspareunie, la sècheresse vaginale et les troubles érectiles du conjoint sont les principaux déterminants de la sexualité à la ménopause.

## Conflits d’intérêts

Les auteurs ne déclarent aucun conflit d'intérêts.
